# LED control of gene expression in a nanobiosystem composed of metallic nanoparticles and a genetically modified *E. coli* strain

**DOI:** 10.1186/s12951-021-00937-x

**Published:** 2021-06-26

**Authors:** Hossein Alishah Aratboni, Nahid Rafiei, Larousse Khosravi Khorashad, Albert Isaac Lerma-Escalera, Francisco de Jesús Balderas-Cisneros, Zhaowei Liu, Abbas Alemzadeh, Sadasivan Shaji, José Ruben Morones-Ramírez

**Affiliations:** 1 Universidad Autónoma de Nuevo León, UANL. Facultad de Ciencias Químicas, Av. Universidad s/n. CD. Universitaria, San Nicolás de los Garza, 66451 Nuevo León México; 2 Centro de Investigación en Biotecnología y Nanotecnología, Facultad de Ciencias Químicas, Universidad Autónoma de Nuevo León. Parque de Investigación e Innovación Tecnológica, Km. 10 autopista al Aeropuerto Internacional Mariano Escobedo, 66629 Apodaca, Nuevo León México; 3 Department of Crop Production and Plant Breeding, School of Agriculture, Shiraz University, Km. 12 Shiraz-Isfahan highway, Bajgah area, 71441-65186 Shiraz, Iran; 4 Department of Electrical and Computer Engineering, University of California, San Diego, 9500 Gilman Drive, La Jolla, CA 92093 USA; 5 Universidad Autónoma de Nuevo León, UANL. Facultad de ingeniería mecánica y eléctrica, Universidad s/n. CD. Universitaria, 66451 Nuevo León San Nicolás de los Garza, México

**Keywords:** Nanobiosystem, Gold nanoparticles, Mathematical model opto-thermal conversion, Opto-thermal nanoconverters, Metallic nanoparticles, Bioprocess production, Opto-thermal gene expression

## Abstract

**Background:**

Within the last decade, genetic engineering and synthetic biology have revolutionized society´s ability to mass-produce complex biological products within genetically-modified microorganisms containing elegantly designed genetic circuitry. However, many challenges still exist in developing bioproduction processes involving genetically modified microorganisms with complex or multiple gene circuits. These challenges include the development of external gene expression regulation methods with the following characteristics: spatial–temporal control and scalability, while inducing minimal permanent or irreversible system-wide conditions. Different stimuli have been used to control gene expression and mitigate these challenges, and they can be characterized by the effect they produce in the culture media conditions. Invasive stimuli that cause permanent, irreversible changes (pH and chemical inducers), non-invasive stimuli that cause partially reversible changes (temperature), and non-invasive stimuli that cause reversible changes in the media conditions (ultrasound, magnetic fields, and light).

**Methods:**

Opto-control of gene expression is a non-invasive external trigger that complies with most of the desired characteristics of an external control system. However, the disadvantage relies on the design of the biological photoreceptors and the necessity to design them to respond to a different wavelength for every bioprocess needed to be controlled or regulated in the microorganism. Therefore, this work proposes using biocompatible metallic nanoparticles as external controllers of gene expression, based on their ability to convert light into heat and the capacity of nanotechnology to easily design a wide array of nanostructures capable of absorbing light at different wavelengths and inducing plasmonic photothermal heating.

**Results:**

Here, we designed a nanobiosystem that can be opto-thermally triggered using LED light. The nanobiosystem is composed of biocompatible gold nanoparticles and a genetically modified *E. coli* with a plasmid that allows mCherry fluorescent protein production at 37 °C in response to an RNA thermometer.

**Conclusions:**

The LED-triggered photothermal protein production system here designed offers a new, cheaper, scalable switchable method, non-destructive for living organisms, and contribute toward the evolution of bioprocess production systems.
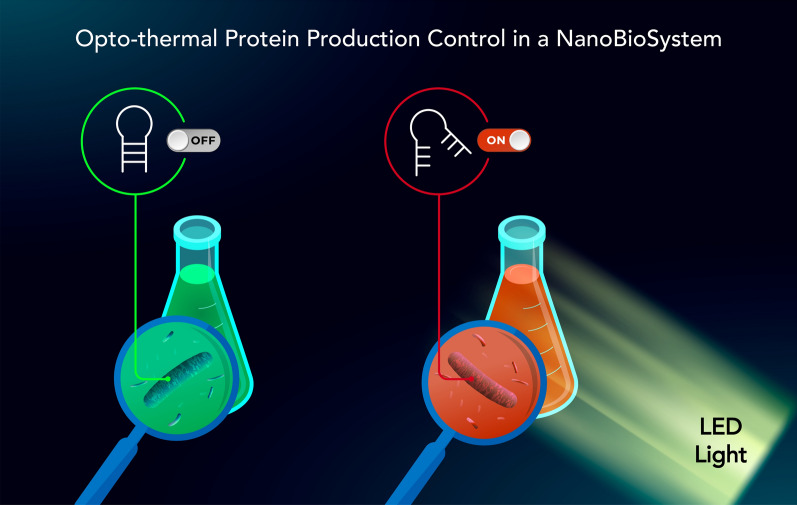

**Supplementary Information:**

The online version contains supplementary material available at 10.1186/s12951-021-00937-x.

## Introduction

One of the many challenges in the development of engineered biological systems is to control gene expression [1]. Since most genes have a specific biological function, it is of interest to develop engineered biological systems that control both spatially and temporarily, the on and off state of gene expression [2]. Control of gene expression can occur at both the transcription and the post-transcription stages [3]; however, one of the most common is post-transcription gene regulation, which refers to controlling the translation of mRNA into a protein [4]. In living cells, various mechanisms have been developed and engineered to control gene expression; mechanisms can be through external stimuli such as changes in temperature [5] or induction by light, or internal stimuli such as changes in the environmental pH [6], the use of chemical inducers [7] and changes in other culture conditions [8]. However, although very efficient, the internal induction mechanisms usually induce an irreversible on–off state, and the external mechanisms such as temperature are usually slowly reversible and lack space control specificity. Therefore, much research has been focused on working with light-controlled gene expression since light is a stimulus that induces fast-switching, non-invasive gene expression mechanisms with a highly space-specificity, especially when working with lasers. [9, 10]. Light can be naturally absorbed by many photoreceptor proteins found in biological systems such as plants, bacteria, fungi and higher eukaryotes [11]. The structure of these photoreceptor proteins has two distinct parts with specialized roles. The light-sensitive part of these photoreceptor proteins has small molecules called chromophores, which are small molecules capable of receiving light and then transferring energy to another part of these photoreceptor proteins (connected to DNA) [12]. This mechanism for receiving light through chromophores finally leads to changes in the gene expression and, consequently, changes in the transcriptome pattern of the organism [13]. The existence of two major types of electronic and vibrational transitions and the placement of these chromophores together, in order to increase the efficiency [14], leads to the formation of a variety of photoreceptors that can only absorb at a certain light spectrum [15]. However, there are limitations to using these photoreceptors in the engineering of light-controlled gene expression systems. Their response is limited to exposure to specific wavelengths. Therefore, when developing complex gene expression systems, there is a need to incorporate expression to various chromophores, leading in many cases to the instability of these molecules after being stimulated by light [16]. Moreover, some modified fluorescent proteins, such as green fluorescent proteins, based on the same chromophores, in addition to emitting fluorescent light, induce a series of unwanted biochemical side effects such as the production of reactive oxygen species (ROS) [17].

These various limitations associated with the use of chromophores to control gene expression in biological systems led this work to search for alternative systems that would allow the versatility of absorption spectra to develop complex gene expression control. Therefore, this work explored the use of non-toxic metallic nanoparticles, which are opto-thermal energy converter systems capable of interacting with biological systems. Metallic nanoparticles have extraordinary optical properties that differ greatly from those of the bulk macroscopic metal [18]. In specific, gold nanoparticles (AuNPs) present a wide absorption spectrum that can be easily tuned since it directly depends on the size, shape, and surface chemistry of the nanostructure. AuNPs, due to having intense optical absorbance and large absorption cross-sections, can absorb optical energy at a specific wavelength and convert it to thermal energy with high efficiency [19]. When AuNPs are illuminated by incident light, a collective oscillation of free conduction electrons is produced in AuNPs, known as plasmon resonance. A part of the high-energy of the excited plasmon due to phonon− phonon (at a time scale of 100 –380 ps) and electron–phonon (at a time scale of 2–5 ps) interaction can decay in a non-radiative form, producing a large amount of highly localized thermal energy. This process leads to an elevation of the temperature in the surrounding environment of the AuNPs. Then, the generated heat diffuses away from the surface of the hot AuNPs and leads to an increase in the temperature of the surrounding medium [20–24]. Plasmonic photothermal heating using AuNPs has been successfully used for various applications, including; diagnostic reagents [25], drug carriers [26], contrast agents [27], radiosensitizers [25, 28] and specially photothermal therapy agents [29].

Due to the properties of these nanoparticle systems, there have been applications that have been developed to supply optical energy at a specific wavelength, through a laser light, both in a pulse or a continuous wave, in order to stimulate heat-responsive systems, such as free polymers [30] or grafted polymers in porous membranes [31]. However, although very important to control gene expression spatiotemporally, laser light presents a disadvantage in systems that need scalability. Lasers are expensive and need many safety precautions to operate as part of a production system. An additional disadvantage is that lasers can perform only on a small scale, which is one of the challenges addressed in this work.

We here focus on developing a larger scale production system that allows a low-cost, fast-switching gene expression. In our experiments, we developed a biological system that uses a light-emitting diode (LED) instead of a laser device as a new, cheaper, harmless, switchable method, non-destructive for living organisms (at the wavelengths and time periods explored in this work). Specifically, our systems is feasible to operate in a larger scale (to overcome the limitation of laser for large scale studies and operations) to stimulate AuNPs and induce photothermal protein production in a microorganism genetically modified with an RNA thermometer.

RNA thermometers are structurally simple and sensitive to temperature changes. RNA thermometers are thermosensors that can sense temperature changes. They are actually located in the 5’-untranslated region (5´-UTR) of mRNAs and can control the expression of downstream genes by regulating gene expression by temperature-induced changes in RNA conformation [32]. At low temperature (or in other words, in a temperature-dependent manner), the mRNA will mask the ribosome binding site [Shine–Dalgarno (SD) sequence] within the 5 -untranslated region (5´-UTR) and, in this way, prevent ribosome binding and translation. When temperature increases, the RNA secondary structure melts locally. Thereby ribosomes can be joined to the SD sequence to start translation [33]. In this study, by computational design and in vivo screening, we have constructed an RNA element that regulates bacterial gene expression in a temperature-dependent manner. A single small stem-loop structure containing the ribosome binding site, works efficiently at specific temperatures since it melts at a specific temperature and then is translated to a fluorescent protein. Therefore, AuNPs are particularly appealing candidates as switchable temperature sources that can provide the needed temperature changes. The present study has shown that LED can be a promising alternative source instead of a laser device to provide enough temperature (on a large scale) using switchable AuNPs. We here also demonstrate the ability of our designed nanobiosystem to be externally controlled, scalable and completely reversible for future use in bioprocess production systems.

## Results and discussion

### Characterization of the synthesized gold nanoparticles

**Fig. 1:**
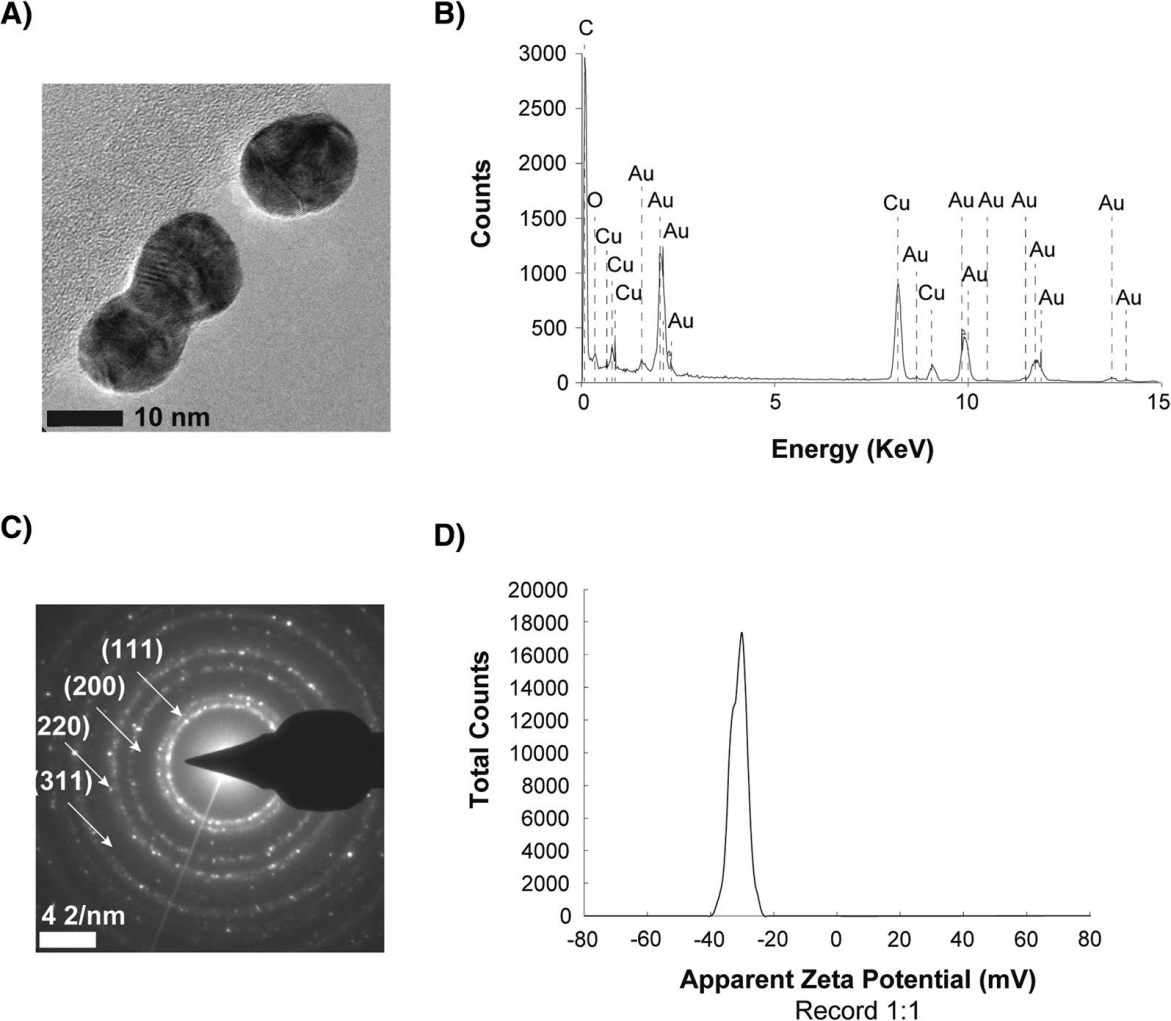
Characterization of synthesized AuNPs. **A** High-resolution Transmission Electron Microscopy image of AuNPs. **B** Energy Dispersive X-ray Spectroscopy spectrum of AuNPs. **C** Selected Area Electron Diffraction pattern of AuNPs and **D** Zeta potential of synthesized AuNPs

Gold nanoparticles (AuNPs) were synthesized through the citrate reduction method, developed by Turkevich et al. [34], and they were physically characterized by high-resolution transmission electron microscopy (HRTEM). HRTEM images show the spherical morphology of the AuNPs and the lattice fringes exhibited by the nanoparticles (Fig. 1A) are shown to correlate to those of gold (Au). The energy-dispersive X-ray spectroscopy (EDXs) spectrum of the AuNPs (Fig. 1B) shows the presence of Au atoms, confirming the composition of the AuNPs. In addition, the EDXs spectrum detects the presence of oxygen (O), corresponding to the oxygen present in the air inside the detection chamber [35] and carbon (C) and copper (Cu) corresponding to the composition of the transmission electron microscopy (TEM) holding grids [36]. Characterization of the AuNPs through selected area electron diffraction (SAED) shows patterns of a single spherical AuNP (Fig. 1C), confirming the single crystalline nature of the AuNPs in the face-centered cubic (FCC) phase. The spotty rings observed (Fig. 1C) correspond to the (111), (200), (220) and (311) planes of the FCC crystalline lattice of AuNPs. The characterization of the AuNPs show zeta potential values of − 34.8 mV (Fig. 1D). This high absolute value of the zeta potential is correlated to highly negative charges on the surface of AuNPs, a direct result of using citrate as a reducing and capping agent in the synthesis of the AuNPs. The high absolute magnitude of the zeta potential exhibited by the synthesized AuNPs is linked to the high stability of the sample since the negative surface charges cause strong repellent forces among the AuNPs and prevent their aggregation and precipitation [37].

**Fig. 2:**
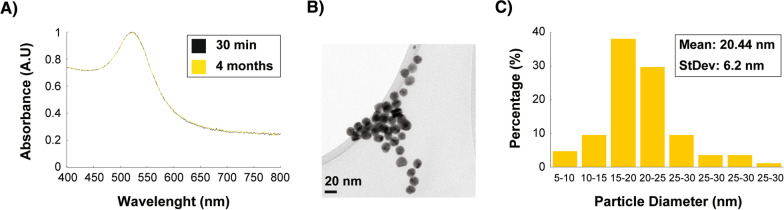
Characterization of synthesized AuNPs. **A** UV–vis absorption spectra of AuNPs; 30 min and 4 months after synthesis of the AuNPs. **B** Transmission electron microscopy image of AuNPs and **C** Particle diameter size distribution of synthesized AuNPs

AuNPs unveil unique optical properties when interacting with specific light wavelengths [38]. We used UV–Vis spectroscopy as a primary characterization technique to measure the surface plasmon resonance (SPR) of the AuNPs. The AuNPs exhibited an optical absorbance around 523 nm, 30 min after the synthesis was completed (Fig. 2A), which correlates with absorption peaks previously reported to be characteristic for AuNPs [39–41]. To monitor the stability of the synthesized AuNPs, their UV–visible absorption spectra were measured and recorded four months after they were synthesized. The absorption peak 30 min after the synthesis does not show a difference with the peak recorded four months after the synthesis (Fig. 2A), demonstrating the stability of the AuNPs sample. The synthesized nanoparticles were morphologically spherical and well dispersed (Fig. 2B). Through the analysis of the diameter size from 800 nanoparticles in different TEM images, the diameter histogram shows the AuNPs to be homogenously dispersed with an average size diameter of 20.44 nm (Fig. 2C).

### Temperature profiles of AuNPs solution when exposed to an LED source

An analysis of variance (ANOVA) was conducted to identify the parameters that significantly affected the induction of a temperature change in a AuNP solution after being exposed to an LED source. The parameters monitored were: AuNPs concentration in the solution, volume of the sample, distance of the sample from the LED source, and exposure time. The results demonstrated that both the individual parameters monitored and their interactions had P-values less than 0.005, indicating their significant effect on the temperature increases exhibited within the AuNPs aqueous solution sample. (Additional file 1: Table S1).

An additional ANOVA was performed when applying a quadratic model. The data, with *x*_*1*_ = concentration, *x*_*2*_ = volume, *x*_*3*_ = distance and *x*_*4*_ = time, showed a correlation coefficient value of 98.42% and an adjusted R^2^ of 96.95% for the fit between the observed and predicted response values. The ANOVA demonstrated that the main effect of all factors, in addition to the interactions of concentration $\times$ distance and time $\times$ distance, in addition to the second-order effects of${{x}_{1}}^{2}$, ${{x}_{3}}^{2}$ and ${{x}_{4}}^{2}$ were significant (Additional file 1: Table S2). Therefore, these factors demonstrated to have a key role in the thermal response of the AuNP solution exposed to the LED source. Based on the following regression model ${Y=\beta }_{0}+\sum {\beta }_{i}{x}_{i}+\sum {\beta }_{ii}{x}_{i}^{2}+\sum {\beta }_{ij}{x}_{i}{x}_{j}$), the ANOVA results in Additional file 1: Table S2, and the regression coefficients, we developed a theoretical model to predict temperature changes in response to the experimental variables measured in the experiments. The equation was fitted as follows:

1 $$Y = 5.42 + 2.47{x_1} + 0.95{x_2} - 3.02{x_3} + 1.2{x_4} - 0.93{x_1}{x_3} - 0.45{x_3}{x_4} + 0.43{x_1}^2 + 1.78{x_3}^2 + 0.31{x_4}^2$$

In Eq. 1, Y represents the temperature change, and x_1_, x_2_, x_3,_ and x_4_ correspond to the concentration of the AuNP solution, the volume, the illumination time of the LED source, and the distance of the AuNP sample from the LED, respectively.

**Fig. 3:**
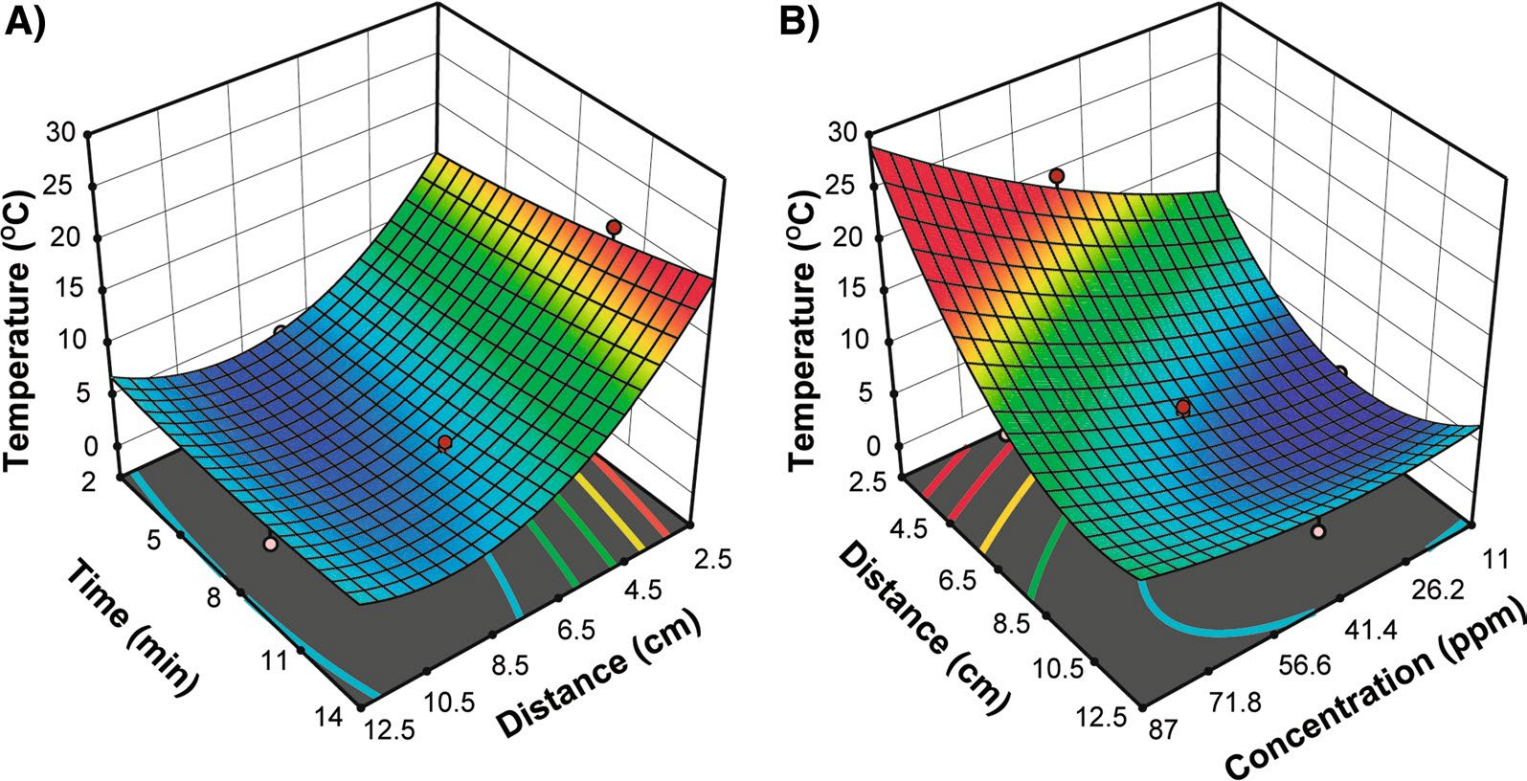
Response surface curve for interaction effect of: **A** Time and distance factors when concentration and volume were maintained at 49 ppm and 1525μL, respectively. **B** Distance and concentration factors when time and volume were maintained at 10 min and 1525μL, respectively

To demonstrate the major interactive effects on the thermal response of a AuNP solution, a 3D surface response plot was constructed (Fig. 3A). In the surface plot the combined effect of time $\times$ distance shows that the temperature of the AuNP solution was enhanced and increased by decreasing the AuNP solution distance from the LED source, due to the negative coefficients observed for distance (Fig. 3A). Furthermore, temperature increases are correlated positively with LED exposition time (Fig. 3A). However, as observed by the parameters of the model, the time factor had a lower effect on the temperature change compared to the distance (Additional file 1: Table S2). An additional positive effect on the thermal response of the AuNP solution was observed for concentration $\times$ distance (Fig. 3B) as the surface plot shows that a maximum can be obtained by decreasing distance and increasing the AuNP concentration in the solution. The concentration of AuNPs has a greater effect of increasing the temperature of the sample, compared to the effect of the illumination time factor (Additional file 1: Table S2). Moreover, the temperature in the samples is dramatically decreased when the concentration of AuNPs is decreased in the sample and the distance is maintained constant (Fig. 3B). A similar effect is observed, but in a lower magnitude, when illumination time is decreased and the distance is maintained at a constant value (Fig. 3A).

To validate the ability of the proposed theoretical regression model to predict the experimental temperature changes, we tested the experimental test parameters (44 ppm concentration, 2800μL AuNPs, 10 min illumination, and distance = 5 cm), and the experimental value showed a ∆T = 12 °C. The proposed theoretical regression model (Eq. 1) predicted a ∆T = 11.8 °C; this shows that the proposed model was in agreement with experimental results and suggests that the proposed theoretical model can be utilized to predict temperature changes observed in the designed system.

Next, by plane electromagnetic wave excitation, we calculated the cross-sections of absorption, scattering, and extinction of the AuNPs using the average of 20.44 nm diameter, obtained from our synthesized particles. The analytical solution of the scattering, for simple spheres, was calculated using Mie theory. By combining this, with the Drude model and the experimental bulk permittivity of gold [42], we incorporated the intrinsic broadening of the absorption spectrum of our sample (Additional file 1: Figure S1). The Drude parameters used were ${\Gamma _{bulk,D}} = 0.076eV$ and ${\omega _p} = 8.9\;eV$, where ${\mathrm{\Gamma }}_{bulk,D}$ and ${\omega }_{p}$ corresponding to the broadening of the absorption spectrum and the plasma frequency, respectively. The experimental bulk permittivity of gold has been taken from the literature [43] and the relative permittivity of water used was ${\varepsilon }_{water}=1.8.$

We next considered the time-dependent heat dissipation resulted from the ensemble of AuNPs in an aqueous solution based upon the absorption of one AuNP. Assuming an absence of convection, the time-dependent heat equation used was:

2 $$\rho \left(\overrightarrow{r}\right)c\left(\overrightarrow{r}\right)\frac{\partial T\left(\overrightarrow{r},t\right)}{\partial t}=\overrightarrow{\nabla }\bullet \left(k\left(\overrightarrow{r}\right)\overrightarrow{\nabla }T(\overrightarrow{r},t)\right)+{Q}_{heating}\left(\overrightarrow{r},t\right)=0.$$

For Eq. 2, $\overrightarrow{r}$ represents the local position, $t$ corresponds to the time and $T$ to the local temperature dependent of time. The local thermal properties are defined as thermal conductivity, $k$, density, $\rho$, and specific heat capacity, $c$. In addition, the ${Q}_{heating}$ represents the local collective volumetric heat flux corresponding to the resistive losses in the system. In our proposed model, the resistive losses only come from the AuNPs since gold is the only material with a non-zero imaginary part for the permittivity. In the ensemble of metal nanoparticles, ${Q}_{heating}$ can be defined as follows [44]:

3 $${Q}_{heating}\left(\overrightarrow{r}\right)={\rho }_{AuNP}\left(\overrightarrow{r}\right)\bullet {\sigma }_{abs-AuNP}\bullet {I}_{LED}\left(\overrightarrow{r}\right).$$

For Eq.  3, ${\rho }_{AuNP}$ corresponds to the number density of AuNPs in solution, ${\sigma }_{abs-AuNP}$ is the absorption cross-section of AuNP, and ${I}_{LED}$ is the local LED intensity. In the illuminating experiment, the solution is at a distance $x$ and, since the LED light is diverging, we approximate the LED power reduction at the position of the ensemble solution by the inverse square law of light as ${I}_{LED}\left(\overrightarrow{r}\equiv \overrightarrow{x}\right)\propto 1/{(4\pi x}^{2}).$ Moreover, the light intensity decays in the absorptive solution according to the Beer-Lambert Law, therefore, the intensity of LED decays in the solution proportional to $\mathrm{e}\mathrm{x}\mathrm{p}(-\alpha r)$ where $\alpha$ is the absorbance decay constant. Equation 3 was used in order to predict the behavior of our illumination-heating experiments by incorporating the following parameters: $d = 5\;cm,\;{\rho _{AuNP}} = 3.26 \times {10^{17}}\;1/{m^3},\;{\sigma _{abs - AuNP}} = 3.46 \times {10^{ - 16}}\;{m^2}$ at wavelength $522nm$, and ${P_{LED}} = 50\;W$ where ${P}_{LED}$ is the LED power at $\overrightarrow{x}=0.$ The AuNPs number density of $3.26 \times {10^{17}}\;1/{m^3}$ corresponds to a 44 ppm concentration of AuNPs in solution. In the experimental setup, a cuvette with cross section area of $10mm\times 12.2mm$ is filled with a 2,800µL solution of AuNPs. We approximated the cuvette as a rectangular prism filled up to 2.24 cm. The entire system is in contact with air at ambient temperature. The time-dependent calculations of the temperature are displayed for two different concentrations, 44 ppm and 88 ppm (Additional file 1: Figure S2). It can be observed that the temperature increases linearly at early times and then reaches a steady state at longer exposure times (hours). The inset of Additional file 1: Figure S2 demonstrates that linear behavior approximation can be used at early illumination times (less than 25 min). Moreover, the calculation for local collective heating ${Q}_{heating}\left(\overrightarrow{r}\right)$ of a 44 ppm AuNP aqueous sample, after exposure to the LED in $+x$ direction shows that heat decays following the Beer-Lambert Law and the inverse square law along the $x$ axis (Additional file 1: Figure S3A). In addition, for the temperature profile along the x-axis (Additional file 1: Figure S3B), going through the middle of the cuvette, a linear temperature profile is observed inside the solution, for LED exposure times less than 1 h. The temperature differences between both opposite walls of the cuvette are between 1 and 2 °C (Additional file 1: Figure S3B).

**Fig. 4:**
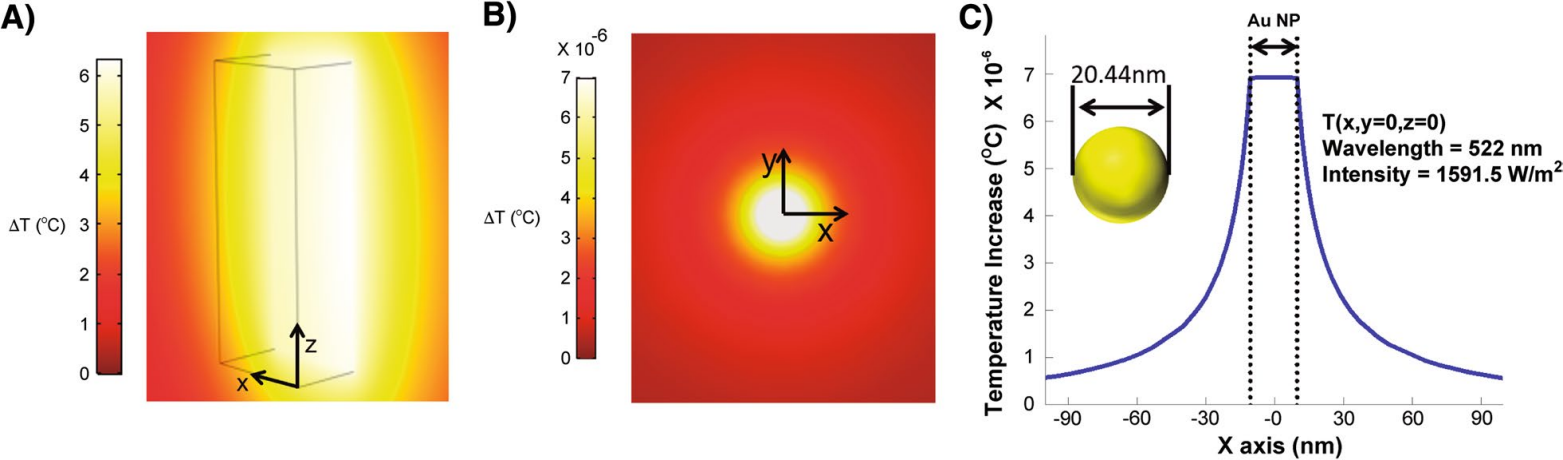
**A** Temperature distribution along $xz$ plane at $y=11.2mm$ at *t* = 10 min. **B** Steady state temperature distribution of one AuNP in water at wavelength $522nm$ with diameters 20.44 nm. **C** Temperature profile of AuNP along $x$ axis for the same intensity of LED applied to the ensemble of AuNPs. Inset shows the geometry of AuNP

To compare the theoretical and experimental approaches, we again used the experimental test parameters (44 ppm concentration, 2800μL AuNPs, 10 min illumination, and distance = 5 cm) that results in a temperature change of ∆T = 12 °C. The theoretical simulation of the temperature distribution at $t=10min$ along the $xz$ plane showed that within the geometry of the cuvette, shown with three-dimensional black lines, the maximum temperature achieved inside the solution is ~ 12.7 °C which is in agreement with our experimental observations (Fig. 4A). The simulation result represents the maximum temperature resulting from the local collective heat derived from the maximum heat dissipated from the surface of the AuNPs (Fig. 4B, C).

### Regulation of mCherry protein production in E. coli cloned with a genetic circuit regulated with an RNA thermometer

An *Escherichia coli* DH5α strain (*E. coli* DH5α) was cloned with an mCherry producing plasmid regulated by a U6 RNA thermometer. The cloned *Escherichia coli* DH5α strain (*E. coli* cDH5α) colonies, were selected using LB agar with kanamycin as a selection marker. The *E. coli* cDH5α strain was cultured overnight in LB medium at both 37 °C and 30 °C. After 16 h of incubation, the cultures were centrifuged. The observed reddish color of the bacterial pellet confirmed that the *E. coli* cDH5α strain was able to produce the mCherry protein when it was grown at 37 °C (Additional file 1: Figure S4 (right)). When the *E. coli* cDH5α strain was grown at 30 °C, no mCherry protein production was expressed (Additional file 1: Figure S4 (left)).

#### Photothermal protein production induced by LED Exposure in *E. coli* DH5α

**Fig. 5:**
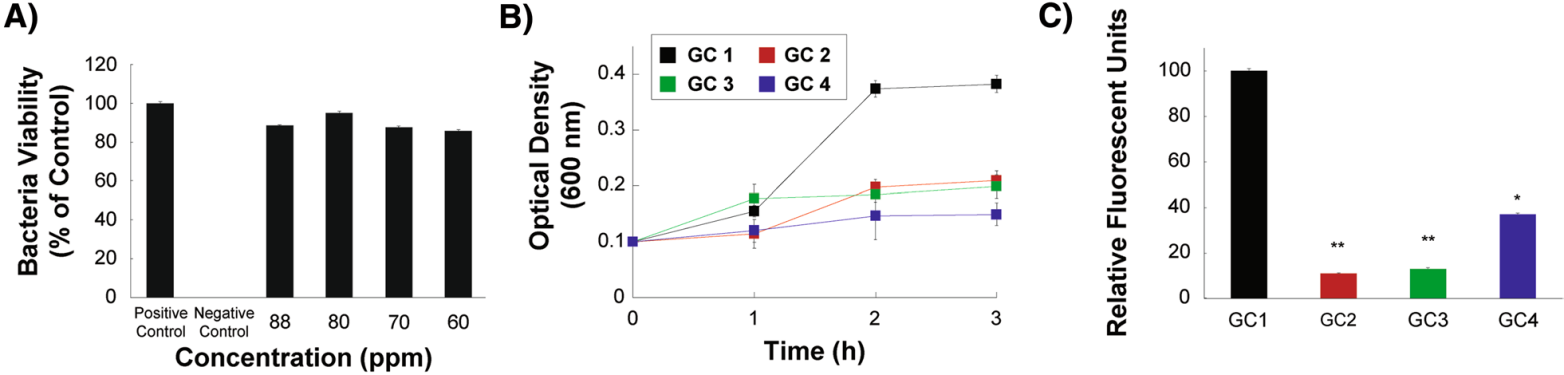
Photothermal protein production. **A** Toxicity effect of different concentrations of AuNPs on *Escherichia coli* cDH5α. **B** Optical density growth curves of *Escherichia coli* cDH5α under the following growth conditions; *GC 1* presence of AuNPs and in the presence of LED light illumination; *GC 2* in the absence of AuNPs and in the presence of LED light illumination; *GC 3* in the presence of AuNPs and in the absence of LED light illumination; *GC 4* in the absence of AuNPs and in the absence of LED light illumination. **C** Normalized Relative fluorescence units (RFU) of the *mCherry* proteins produced after 3 h growth under the different growth conditions (GC 1, GC 2, GC 3 and GC 4) in the *Escherichia coli* DH5α cloned with the mCherry producing plasmid. Data are expressed as mean ± SEM, n = 3 well; *P $<$ 0.05 and **P $<$ 0.01 vs. S1

We built the photothermal triggered protein production nanobiosystem composed of metallic nanoparticles, which convert the LED source into heat, and the cloned *E. coli* strain (*E. coli* cDH5α) that contains the U6 RNA thermometer that regulates the mCherry protein production in response to heat. We tested the nanobiosystem for biocompatibility by exposing the *E. coli* cDH5α strain to different concentrations of AuNPs to assess their toxicity. As observed from our results, after exposure to concentrations of AuNPs between 60 and 88 ppm, the data are not statistically different from the positive control, confirming that the AuNPs do not show toxicity or an antimicrobial effect against our *E. coli* cDH5α strain (Fig. 5A). These results are consistent with previous literature where gold nanoparticles have not been reported to exhibit a toxic effect or growth inhibition against *E. coli* strains [41].

*E. coli* cDH5α was grown at ambient temperature under four different growth conditions (GC): GC 1, in the presence of both AuNPs and LED light illumination; GC 2, in the absence of AuNPs and in the presence of LED light illumination; GC 3 in the presence of AuNPs and in the absence of LED light illumination; and GC 4 in the absence of AuNPs and in the absence of LED light illumination. The optical density (OD) measurements demonstrated that only when the growth conditions involved the exposure to LED illumination and the presence of AuNPs, *E. coli* cDH5α could grow exponentially and reach a bacterial growth OD_600nm_ of 0.37 within the first 2 h. Moreover, the *E. coli* cDH5α culture reaches a stationary phase, and the OD_600nm_ at 3 h is maintained constant (Fig. 5B). The rest of the culture growth conditions showed only slight increases in the *E. coli* cDH5α growth within the first 3 h, and significantly lower than those observed in the culture conditions involving both the presence of AuNPs and exposure to illumination (Fig. 5B). This phenomenon is observed since the heating of the sample allows reaching a higher temperature, and a more optimal condition, during illumination and the presence of the AuNPs, compared to the other growth conditions. This temperature increase is achieved since the presence of the AuNPs induce heating through their opto-thermal converter properties.

After 3 h of *E. coli* cDH5α growth under the different culture conditions, the relative fluorescence was measured and normalized per bacterial cell. As shown in Fig. 5C, production of the mCherry protein is only prominently observed at growth conditions where both AuNPs and LED illumination is present. The presence of both of these factors induce the expression of the mCherry protein; since the generated heat enables the opening of the stem-loop structure containing the ribosome binding site and consequently leads to the production of the mCherry protein. However, the stem-loop structure is not disrupted significantly under the other growth conditions, leading to a low expression of the mCherry protein for all of the other growth conditions. Altogether these results demonstrate the ability of our NanoBioSystem to optically control and trigger gene expression in the biological sample, for the case composed of an *E. coli* DH5α strain cloned with an RNA thermoregulator, through nanoparticles which act as opto-thermal energy converters.

## Conclusions

Achieving adequate control of biological processes is an essential strategy in developing and optimizing the biosynthesis of a wide variety of products with applications in different industrial sectors. Specifically, photothermal regulation is an attractive option for optimizing many biological processes, as it is non-invasive, fast-responsive, and reversible. In this work, we established a promising nanobiological system composed of AuNPs and a genetically modified microorganism with an RNA thermometer that regulates the expression of a fluorescent protein. The characteristics of the components in the nanobiosystem allow the use of a LED to control gene expression and photothermally induce the production of a fluorescent protein. LED is a scalable, cheap, harmless, switchable external stimuli, in addition to non-toxic for living organisms. Therefore, based on the results reported here, we consider the nanobiosystem here developed represents a highly relevant modular gene expression system with a wide array of applications in the design and development of complex bioprocesses. Moreover, LED external expression systems can be further explored to control gene expression in other prokaryotic and eukaryotic cells.

## Methods

### Synthesis of gold nanoparticles

The synthesis of the gold nanoparticles (AuNPs) was carried out using the Turkevich method, also known as the citrate reduction method [34]. The synthesis involved preparing 300 mL of 0.5 mM aqueous gold chloride solution in a 1L beaker, followed by boiling the solution on a hot plate and sitting the solution at 600 rpm until boiling. As soon as boiling was observed, 30 mL of aqueous 38.8 mM trisodium citrate solution was added to the boiling solution in the beaker. Immediately after adding the trisodium citrate solution, the reaction in the beaker turned from a colorless solution to a dark, violet-red color, followed by turning maroon. The final product of the reaction, the synthesized gold nanoparticle solution, was brought to room temperature to cool down, and the solution then turned from maroon to a red color.

### Characterization of gold nanoparticles

Transmission electron microscopy (TEM) and selected area electron diffraction (SAED) were employed in an FEI-TITAN 80–300 kV transmission electron microscope operated at an accelerating voltage of 300 kV to analyze and characterize the AuNPs shape, average size, particle size distribution, and crystalline structure. The elemental composition of the gold nanoparticles was analyzed through an energy-dispersive X-ray spectroscopy (EDS) analyzer integrated into the FEI-TITAN 80–300 kV transmission electron microscope. The AuNPs were also characterized using a zetasizer (Nano ZS90 model) to measure the zeta-potential of the synthesized AuNPs and evaluate the suspension stability.

### Set-up and assessment of LED chip suitability and feasibility for large scale use

To set-up and assess the LED chip suitability and feasibility for larger scale use, the green LED chip, with a peak wavelength of 520–525 nm and a viewing angle of $140^\circ$ was mounted onto a heat sink with an incorporated cooling fan of the same size, driven only by electric power (Additional file 1: Figure S5). LED potency and its functional capability to stimulate the aqueous suspension of AuNPs and induce a temperature profile were assessed by exposing semi-micro UV-cuvettes (transparent to 230–900 nm wavelengths), containing different concentrations and volumes of AuNPs solutions, to the LED source. The LED light was directly illuminated on the cuvette at different distances from the samples during a range of illumination times. Temperature changes were immediately measured and recorded using a professional, sensitive RMS Digital Multimeter sensor.

### Experimental temperature profile

To identify and study the effective variability and temperature differences reached within the AuNP solutions, a statistical approach was employed. First, to screening the various factors that induced a significant effect on the temperature changes in the AuNPs, a full factorial design was used employing the design expert software (version 11.0.5, STAT-EASE, Minneapolis, MN, USA). The research was carried out considering four factors at three different levels (Additional file 1: Table S3): concentration and volume of the AuNP solution, time of light illumination, and distance of AuNP solution from the LED light source.

Following screening and identifying the significant factors, to achieve an optimal AuNP photothermal treatment, Central Composite Design (CCD) and Response Surface Methodology (RSM) were employed, based on mathematical and statistical techniques. The relationship between the response, the independent variables, and the optimization of significant factors (time illumination of light, the distance of AuNP container from LED, concentration, and volume of AuNP solution) were explored. These four factors were investigated at five different levels, and a set of 81 experiments were accomplished. After the tests were completed, statistical methods were used to analyze the experimental data. Design-Expert software was used to perform the design and to analyze the experiment. The mathematical relationship between the temperature change as a dependent variable, and the other independent variables, was described using the following second-order polynomial model:

4 $${Y=\beta }_{0}+\sum {\beta }_{i}{x}_{i}+\sum {\beta }_{ii}{x}_{i}^{2}+\sum {\beta }_{ij}{x}_{i}{x}_{j}$$

where $Y$ was the dependent or predicted response, ${x}_{i}$ and ${x}_{j}$ were the coded independent factors,${\beta }_{0}$ was the interception coefficient; ${\beta }_{i}$ was the linear coefficient and ${\beta }_{ii}$ and ${\beta }_{ij}$ were the quadratic and interaction coefficients respectively. In order to illustrate the major interactive effects on thermal response of AuNP solution, a 3D response surface plot was employed.

### Theory and simulation

To compare the theoretical and experimental approaches, we simulated heat generation from our AuNPs. Because AuNPs have specific characteristics depending on their morphology and size, a simulation was performed to predict the light-matter interaction with the gold nanoparticles (AuNPs) and the heat generated from the AuNP ensemble using the finite element method and the software COMSOL Multiphysics.

### Design of genetic temperature sensor and construction of genetically modified *E. coli*

**Fig. 6:**
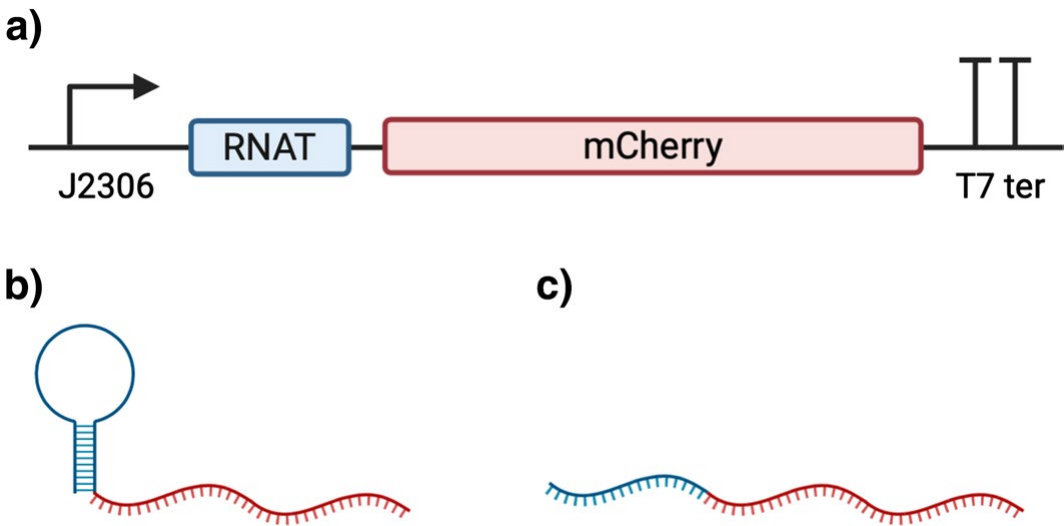
Genetic temperature sensor structure; The constitutive promotor BBa_J23119 for a constant production of RNA, the U6 RNA thermometer conformed by a Shine-Dalgarno (SD) sequence, an anti-SD sequence, and a spacer sequence, the mCherry protein as reporter with a LVA tag to avoid the *mCherry* accumulation, and a T7 transcriptional terminator

A temperature biosensor capable of controlling gene expression was built through the design of a synthetic genetic circuit conformed of a BBa_J23106 constitutive promoter, a U6 RNA thermometer, and the codified sequence of an mCherry protein tagged with LVA, to avoid mCherry accumulation, and a T7 transcription terminator (Fig. 6). The nucleotide sequence of the constructed sensor is described in Additional file 1: Figure S6. The U6 RNA thermometer[32] used in the constructed and designed synthetic gene circuit has been previously used and the characteristics of the thermosensor has been reported to have an optimal on state temperature of 37 °C, where the mCherry protein will be produced, and an off state temperature below 30 °C, where there would be no protein production[32]. The mCherry protein was used as the reporter protein. This monomeric protein can be measured by fluorescence, (with a peak fluorescent excitation and emission at 587 nm and 610 nm, respectively), has a maturation time of 15 min [45] and its labeled with a LVA degradation tag for a constant degradation. The mCherry producing gene, linked to the U6 RNA thermometer, was synthesized and cloned in a pUC57-Kan plasmid by the commercial company BioBasic. The lyophilized synthesized plasmid (pUC57-U6mCherryLVA) was transformed using an *Escherichia coli* DH5α electro-competent strain [46]. Colonies were selected using LB agar with kanamycin and the red-phenotype of the colonies as selection markers.

### Photothermal protein production by LED in *E. coli* DH5α cloned strain

Since we intended to use the *Escherichia coli* cDH5α strain in the photothermal protein production application, toxicity effects of the AuNPs where tested at different concentrations (60, 70, 80 and 88 ppm). The standard broth micro-dilution (BMD) method using 96-well microtiter plates [47] was performed as it was recommended by the Clinical and Laboratory Standards Institute (CLSI) to detect the sensitivity of the standard strains into AuNPs, with a minimum inhibitory concentration (MIC). Briefly, *E. coli* cDH5α strain cells were sampled from a bacteria pre-cultured medium (20µL) and were grown in Luria–Bertani (LB) medium (5 mL) with 5µL kanamycin (50 mg/mL) at 37 °C under continuous shaking (150 rpm). Then, AuNPs were dissolved in water and different concentrations of AuNPs were tested (60, 70, 80 and 88 ppm). Luria–Bertani medium as a negative control were also prepared. 100µL of different concentration of AuNPs diluted in Luria–Bertani (100µL) containing the *E. coli* cDH5α strain suspension (1 $\times$ 10^8^ CFU/mL) and then incubated at 37 °C for 24 h. The bacterial growth was measured as turbidity with a Multiskan™ GO Microplate Spectrophotometer at an OD of 600 nm. The results were then analyzed based on a two-sample student’s t-test to determine statistical differences between the treatments and our positive control grown without the presence of AuNPs.

The previous literature has reported that the 6U RNA thermometer can be provisionally opened and work efficiently at 37 °C. Beside, achieved temperature profile and related analysis in last section have showed that we can obtain a given specific absorption rate (SAR) and thermal response in solution (it mean bacteria surrounding) and finally achieve optimal AuNPs (GNP) photothermal protein production through changing and regulation of main effective factors such as distance of LED light source and intended sample and also select and apply the appropriate concentration of AuNPs, volume of solution and illumination time. Based on our achieved regression model (Eq. 1) and analysis of response surface curve (Fig. 3) we found that intended temperature (37 °C) in 2800µL of AuNPs (with final concentration of 44 ppm) can be obtained in distance of 5 cm. In the next step, in order to examination of LED source light, thermal response of AuNPs, stem-loop opening and finally efficiently of photothermal protein production, our experiment is done in two different condition including under illumination and without illumination which each one of them had two experimental sample. Under illumination; Sample 1) a cuvette containing the 1400µL AuNPs (with final concentration of 44 ppm) and 1400µL *E. coli* cDH5α strains (with final OD = 0.1) as main test and also Sample 2) a cuvette contain only 2800µL *E. coli* cDH5α strains as control. Without illumination; Sample 3 and Sample 4 which their content was exactly the same as the sample 1 and 2 respectively. Optical density (OD) measurements were recorded at 600 nm using a UV–Vis spectrophotometer (LMAI-1-ES-3) every 1 h and then growth curves were generated for each one of samples.

Because the bacterial cells, during their exponential phase exhibit their maximum growth rate, relative fluorescence units (RFU) related to mCherry protein production of in this phase with three repetitions, excitation and emission wavelength of 587 and 610 nm respectively were quantified for each one of samples using Microplate Readers (Thermo Scientific™ Varioskan™ LUX) and SkanIt™ Software. Also, relative fluorescence units (RFU) of produced *mCherry* protein in their exponential phase were quantified**.**

## Supplementary Information

**Additional file 1:** Figures and Tables.

## Data Availability

Data sharing is not applicable to this article as no datasets were generated or analyzed during the current study.
