# Correction to: LED control of gene expression in a nanobiosystem composed of metallic nanoparticles and a genetically modifed *E. coli* strain

**DOI:** 10.1186/s12951-022-01293-0

**Published:** 2022-02-17

**Authors:** Hossein Alishah Aratboni, Nahid Rafei, Larousse Khosravi Khorashad, Albert Isaac Lerma-Escalera, Francisco de Jesús Balderas-Cisneros, Zhaowei Liu, Abbas Alemzadeh, Sadasivan Shaji, José Ruben Morones-Ramírez

**Affiliations:** 1 Universidad Autónoma de Nuevo León, UANL. Facultad de Ciencias Químicas, Av. Universidad s/n. CD. Universitaria, San Nicolás de los Garza 66451, Nuevo León, México; 2 Centro de Investigación en Biotecnología Y Nanotecnología, Facultad de Ciencias Químicas, Universidad Autónoma de Nuevo León, Parque de Investigación E Innovación Tecnológica, Km. 10 autopista al Aeropuerto Internacional Mariano Escobedo, 66629 Apodaca, Nuevo León México; 3 Department of Crop Production and Plant Breeding, School of Agriculture, Shiraz University, Km. 12 Shiraz-Isfahan highway, Bajgah area, 71441-65186 Shi-raz, Iran; 4 Department of Electrical and Computer Engineering, University of California, San Diego, 9500 Gilman Drive, La Jolla, CA 92093 USA; 5 Univer-Sidad Autónoma de Nuevo León, UANL. Facultad de Ingeniería Mecánica Y Eléctrica, Universidad S/N. CD. Universitaria, 66451 San Nicolás de los Garza, Nuevo León México

## Correction to: J Nanobiotechnol (2021) 19:190 https://doi.org/10.1186/s12951-021-00937-x

Following publication of the original article [1], one of the corresponding authors e-mail address is omitted from the author group.

The email address of the corresponding author, Abbas Alemzadeh, is alemzadeh@shirazu.ac.ir.

The original article [1] has been corrected.
