# Supplementary material for: LED control of gene expression in a nanobiosystem composed of metallic nanoparticles and a genetically modified E. coli strain
Source: J Nanobiotechnology. 2021 Jun 26;19:190. doi: 10.1186/s12951-021-00937-x (PMC8236197; doi:10.1186/s12951-021-00937-x)
Supplement: Supplementary file 1 — Additional file 1: Figures and Tables. [file 12951_2021_937_MOESM1_ESM.pdf]

LED control of Gene Expression in a NanoBioSystem Composed of Metallic Nanoparticles and a Genetically Modified *E. coli* strain

Hossein Alishah Aratboni[a,b, Ψ], Nahid Rafiei[a,b,c, Ψ], Larousse Khosravi Khorashad[d], Albert Isaac Lerma-Escalera[a,b], Francisco de Jesús Balderas Cisneros[a,b], Zhaowei Liu[d], Abbas Alemzadeh[c,*], Sadasivan Shaji[e], José Rubén Morones Ramírez[a,b,*]

[a] *Universidad Autónoma de Nuevo León, UANL. Facultad de Ciencias Químicas. Av. Universidad s/n. CD. Universitaria, 66451, San Nicolás de los Garza, NL, México.*

[b] *Centro de Investigación en Biotecnología y Nanotecnología, Facultad de Ciencias Químicas, Universidad Autónoma de Nuevo León. Parque de Investigación e Innovación Tecnológica, Km. 10 autopista al Aeropuerto Internacional Mariano Escobedo, 66629, Apodaca, Nuevo León.*

[c] *Department of Crop Production and Plant Breeding, School of Agriculture, Shiraz University, 71441-65186, Km. 12 Shiraz-Isfahan highway, Bajgah area, Shiraz, Iran*

[d] *Department of Electrical and Computer Engineering, University of California, San Diego, 9500 Gilman Drive, La Jolla, CA 92093, USA.*

[e] *Universidad Autónoma de Nuevo León, UANL. Facultad de ingeniería mecánica y eléctrica. Universidad s/n. CD. Universitaria, 66451, San Nicolás de los Garza, NL, México.*

Ψ Both authors contributed equally to the work

Supplementary Table 1. ANOVA for the experimental parameters of full factorial design.

Variable	SS	df	MS	F-value	p-value
Concentration (X_1)	409.71	2	204.85	247.57	< 0.0001
Volume (X_2)	89.57	2	44.79	54.12	< 0.0001
Distance (X_3)	3283.61	2	1641.80	1984.14	< 0.0001
Time (X_4)	245.49	2	122.74	148.34	< 0.0001
X_1 X_3	89.08	4	22.27	26.91	< 0.0001
X_1 X_4	17.58	4	4.39	5.31	0.0012
X_2 X_3	29.82	4	7.45	9.01	< 0.0001
X_2 X_4	22.44	4	5.61	6.78	0.0002
X_3 X_4	95.87	4	23.97	28.97	< 0.0001

R^2 = 0.99; Adjusted R^2 = 0.98; SS, sum of squares; DF, degrees of freedom and MS, mean square.

P-values less than 0.005 indicate model terms are significant.

Supplementary Table 2. Analysis of variance (ANOVA) for response surface Quadratic model.

Source	SS	df	MS	F-value	p-value	
Model	527.67	14	37.69	66.87	< 0.0001	significant
Concentration (x_1)	146.03	1	146.03	259.07	< 0.0001	
Volume (x_2)	21.66	1	21.66	38.43	< 0.0001	
Distance (x_3)	218.41	1	218.41	387.47	< 0.0001	
Time (x_4)	34.56	1	34.56	61.31	< 0.0001	
x_1x_2	0.4900	1	0.4900	0.8693	0.3659	
x_1x_3	13.69	1	13.69	24.29	0.0002	
x_1x_4	0.4900	1	0.4900	0.8693	0.3659	
x_2x_3	0.6400	1	0.6400	1.14	0.3035	
x_2x_4	0.0400	1	0.0400	0.0710	0.7936	
x_3x_4	3.24	1	3.24	5.75	0.0300	
x_1^2	5.15	1	5.15	9.14	0.0086	
x_2^2	0.9219	1	0.9219	1.64	0.2204	
x_3^2	87.23	1	87.23	154.76	< 0.0001	
x_4^2	2.61	1	2.61	4.63	0.0482	
Residual	8.45	15	0.5637			
Lack of Fit	6.25	10	0.6247	1.41	0.3685	not significant
Pure Error	2.21	5	0.4417			
Cor Total	536.12	29				

Quality of quadratic model	
Correlation coefficient (R^2)	0.9842
Adjusted R^2	0.9695
Predicated R^2	0.9270

Supplementary Figure 1

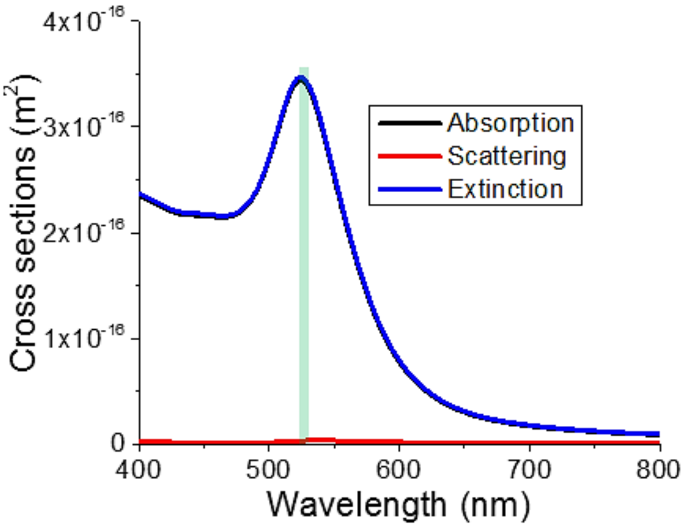

Supplementary Figure 1

Extinction, absorption, and scattering cross sections for a $20.44\ nm$ AuNP in water. The green vertical line shows the LED wavelength excitation range.

Supplementary Figure 2

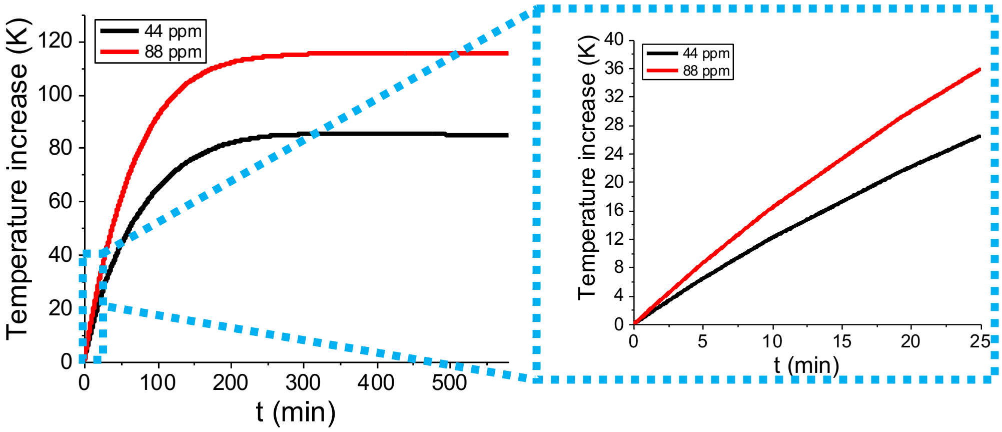

Supplementary Figure 2

Time dependent temperature calculations for two concentrations of 44ppm and 88ppm. The inset is the zoom in plot for early times. It shows linear behavior.

Supplementary Figure 3

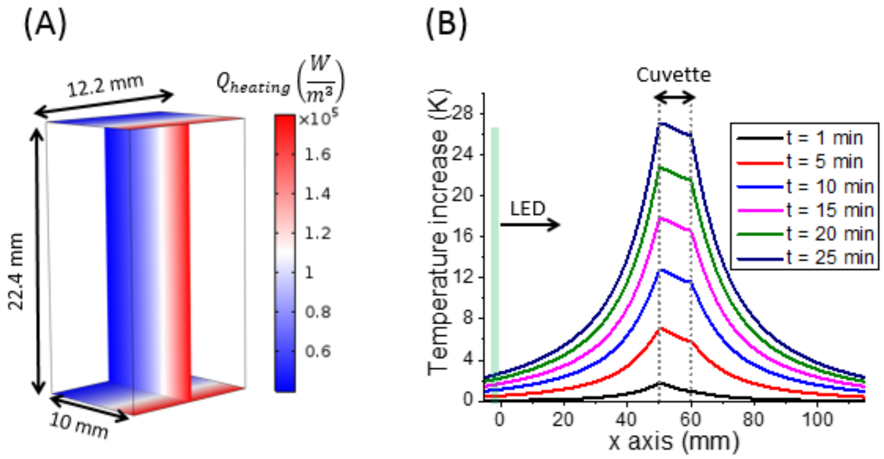

Supplementary Figure 3

(A) Calculated local collective heating of ensemble of AuNPs in the cuvette of 10mm × 12.2mm. The LED excitation is along positive x axis at wavelength of 522nm. **(B)** Temperature profile of the middle of cuvette along x axis for increasing time. The position of LED and cuvette is also shown.

Supplementary Figure 4

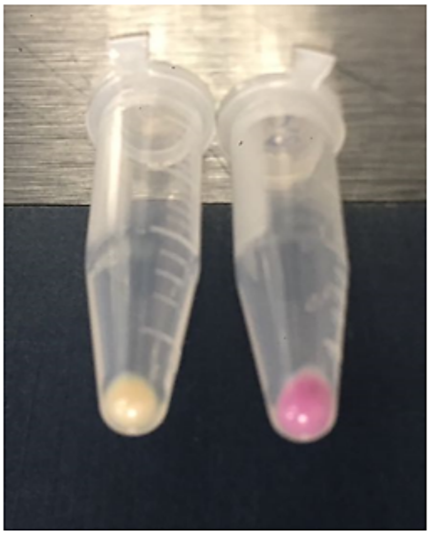

Supplementary Figure 4

Bacterial pellets of *E. coli* DH5α cloned with our sensor after an overnight culture in LB medium at: (right) 37ºC, where the bacteria shows production of the mCherry protein, and at (left) 30ºC where the bacteria does not show production of the mCherry protein.

Supplementary Figure 5

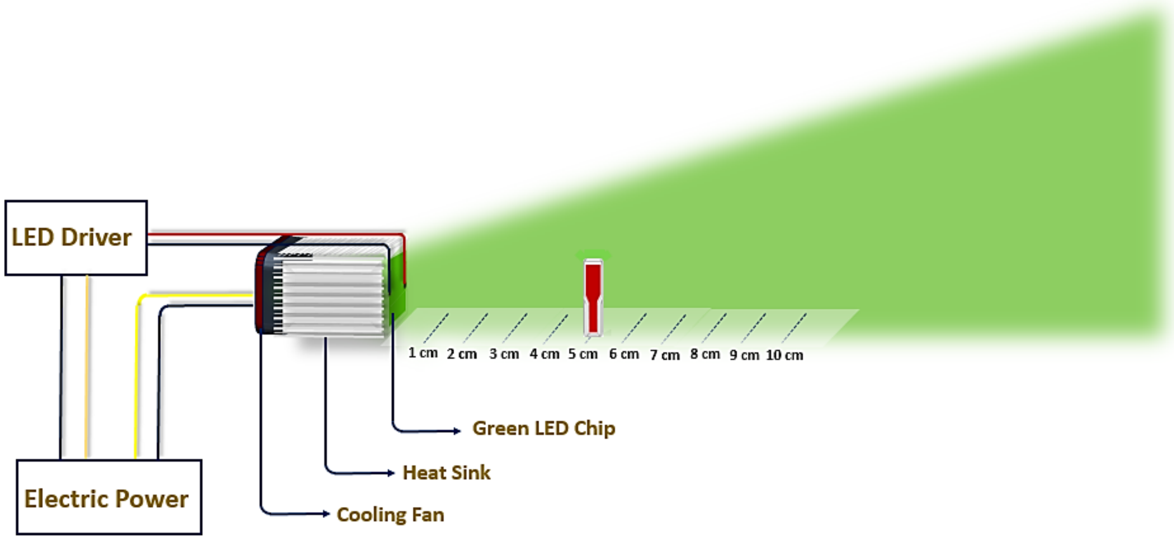

Supplementary Figure 5

Experimental setup for measuring the temperature profile of AuNPs solutions.

Supplementary Table 3

Four experimental variables screened by full factorial design at different levels.

Variable	Units	Levels		
		1	2	3
Time	min	5	10	15
Concentration	ppm	22	44	88
Distance	cm	3	5	10
Volume	μL	200	1400	2800

Supplementary Figure 6

>Temperature sensor sequence

GAATTCGCGGCCGCTTCTAGAGttgacagctagctcagtcctaggtataatgctagcggatccTCTCCTTCa
aaaaaAAAAAAAAAAAAAAAAAAAAAAAAAAAAGGAGATATACCCATGGTTAGCAA
AGGTGAAGAAGATAATATGGCAATTATTAAAGAATTTATGCGTTTTAAAGTTCAT
ATGGAAGGTAGCGTTAATGGTCATGAATTTGAAATTGAAGGTGAAGGTGAAGGT
CGTCCGTATGAAGGTACCCAGACCGCAAAACTGAAAGTTACCAAAGGTGGTCCG
CTGCCGTTTGCATGGGATATTCTGAGCCCGCAGTTTATGTATGGTAGCAAAGCAT
ATGTTAAACATCCGGCAGATATTCCGGATTATCTGAAACTGAGCTTTCCGGAAGG
TTTTAAATGGGAACGTGTTATGAATTTTGAAGATGGTGGTGTTGTTACCGTTACC
CAGGATAGCAGCTTACAGGATGGTGAATTTATTTATAAAGTTAAACTGCGTGGTA
CCAATTTTCCGAGCGATGGTCCGGTTATGCAGAAAAAAACAATGGGTTGGGAAG
CAAGCAGCGAACGTATGTATCCGGAAGATGGTGCACTGAAAGGTGAAATTAAAC
AGCGTCTGAAACTGAAAGATGGTGGTCATTATGATGCAGAAGTTAAAACCACCT
ATAAAGCAAAAAAACCGGTTCAGCTGCCGGGTGCATATAATGTTAATATTAAAC
TGGATATTACCAGCCATAATGAAGATTATACCATTGTTGAACAGTATGAACGTGC
AGAAGGTCGTCATAGCACCGGTGGTATGGATGAACTGTATAAACTGGTTGCATA
AACTAGTAGCGGCCGCTGCAGCATAACCCCTTGGGGCCTCTAAACGGGTCTTGA
GGGGTTTTTGAAGCTT

Supplementary Figure 6

Nucleotide sequence of synthetic temperature sensor.
